# Physical and monetary ecosystem service accounts for Europe: A case study for in-stream nitrogen retention

**DOI:** 10.1016/j.ecoser.2016.11.002

**Published:** 2017-02

**Authors:** Alessandra La Notte, Joachim Maes, Silvana Dalmazzone, Neville D. Crossman, Bruna Grizzetti, Giovanni Bidoglio

**Affiliations:** a European Commission - Joint Research Centre, Directorate D – Sustainable Resources, Via Enrico Fermi 2749, 21027 Ispra, VA, Italy; b Department of Economics and Statistics, University of Torino, Campus Luigi Einaudi, Lungo dora Siena 100, 10153 Torino, Italy; c CSIRO Land and Water Flagship, Waite Campus, 5064 Adelaide, South Australia, Australia

**Keywords:** Q56, Q57, Q25, Ecosystem accounting, Ecosystem services, Water purification, Capacity, Sustainable flow, Actual flow

## Abstract

In this paper we present a case study of integrated ecosystem and economic accounting based on the System of Environmental Economic Accounting — Experimental Ecosystem Accounts (SEEA-EEA). We develop accounts, in physical and monetary terms, for the water purification ecosystem service in Europe over a 20-year time period (1985–2005). The estimation of nitrogen retention is based on the GREEN biophysical model, within which we impose a sustainability threshold to obtain the physical indicators of capacity – the ability of an ecosystem to sustainably supply ecosystem services. Key messages of our paper pertain the notion of capacity, operationalized in accounting terms with reference to individual ecosystem services rather than to the ecosystem as a whole, and intended as the stock that provides the sustainable flow of the service. The study clarifies the difference between sustainable flow and actual flow of the service, which should be calculated jointly so as to enable an assessment of the sustainability of current use of ecosystem services. Finally, by distinguishing the notion of ‘process’ (referred to the ecosystem) from that of ‘capacity’ (pertaining specific services) and proposing a methodology to calculate capacity and flow, we suggest an implementable way to operationalize the SEEA-EEA accounts.

## Highlights

• We present a case study of ecosystem accounting based on the SEEA-EEA framework.
• Accounts for water purification are developed in physical and monetary terms.
• Flow accounts include both actual and sustainable flows.
• Capacity is assessed as Net Present Value of the sustainable flow.
• Replacement cost is the exchange value technique used for the monetary valuation.

## 1 Introduction

Integrated assessments of economic, social and environmental impacts are key to supporting public and private sector decisions related to land and water resources. An essential part of integrated assessments is the identification of the links between ecosystem functions and processes and human wellbeing, a task to which theoretical frameworks, principles, definitions and classifications have been devoted by numerous studies (e.g. Millennium Ecosystem Assessment, 2005; The Economics of Ecosystems and Biodiversity, 2010; Daily et al., 2009; Diaz et al., 2015).

A number of policy initiatives have incorporated ecosystem service quantification and valuation. For example, the *Europe 2020* strategy has the manifest intention of mainstreaming environmental issues into other policy areas (European Commission, 2011a2011, European Commission, 2011b) by preserving the resource base (defined as the capacity of ecosystems to provide services that, in turn, provide benefits to human beings) required to allow the economy and society to function (European Commission, 2011a). The *EU Biodiversity Strategy to 2020* (European Commission, 2011b) includes ecosystem services alongside with biodiversity, to highlight the key role of ecosystems in biodiversity protection. In particular Action 5 of the Strategy requires that ecosystem service assessment and valuation be integrated into accounting and reporting systems, so as to relate environmental assets to other statistics and data on environmental, economic and social characteristics already used by analysts and policy makers. At all levels, a fully integrated economic and environmental analysis is increasingly recognised as crucial for policy design and implementation.

To meet this call, national statistical offices and international agencies have been working on ways to make national accounting and reporting systems more inclusive of ecosystems.^1^ Traditional national economic accounts based on the System of National Accounts (SNA), developed 50 years ago when little thought was given to environmental damage, do not consider ecosystem assets and services. Although there have been some revisions,^2^ the SNA does not yet account for the degradation and depletion^3^ of natural resources. Over the last 40 years a number of efforts have been made to develop methods that integrate traditional macroeconomic indicators with environmental information (Hecht, 2007). In the early 1990s the statistical unit of the United Nations proposed a single System for Integrated Environmental and Economic Accounting (SEEA) (Bartelmus et al., 1991) as a way to standardize different frameworks and methods. The original 1993 SEEA handbook (UN, 1993) focused on the adjustment of existing macro-indicators. The subsequent SEEA 2003 framework comprised four categories of accounts, made up of several environmental accounting modules (UNSD, 2003). More recently, the SEEA Central Framework (SEEA-CF), which covers the main component of the physical environment (air, water and land), is being adopted as an international statistical standard (UN 2014a).

Natural resource accounts, however, only tell part of the story, because ecosystems are a lot more than just land and water. An ecosystem is an interconnected and interacting combination of abiotic (land, water) and biotic (biodiversity) components, and the depletion of its stock - the natural capital - may cause the loss of multiple services now and in the future. This is the reason why ecosystem accounts, aimed at monitoring the capacity of ecosystems to deliver services, are the focus of increasing attention within economic-environmental accounting (Schröter et al., 2014, Remme et al., 2014, Busch et al., 2012, La Notte et al., 2011).

The Land and Ecosystem Accounting framework (LEAC) is an early attempt at ecosystem accounting (Weber, 2009; European Environmental Agency, 2011, European Environmental Agency, 2010, European Environmental Agency, 2006). In LEAC the consumption of natural capital, considered as the asset, is measured as the restoration cost required after intensive exploitation and/or insufficient maintenance. However, the LEAC framework does not incorporate direct measurement of ecosystem services. A white cover version of the SEEA-Experimental Ecosystem Accounts (SEEA-EEA) was released in June 2013 and officially published in 2014 (UN, 2014b), developed and recommended by the United Nations, European Commission, World Bank, OECD and FAO. The SEEA-EEA is an experimental accounting framework to be reviewed in light of country experience and conceptual advances. The framework is intended for ‘multidisciplinary research and testing’ (UN, 2014b) and urgently calls for applications and case studies. SEEA-EEA Technical Guidelines were released in April 2015 and made available for global peer review in December 2015 to support national efforts at ecosystem accounting(UNEP et al. 2015).

The Technical Guidelines state that central in applying the SEEA-EEA framework to ‘support discussion of sustainability’ is the concept of *capacity* (ref. Section 7.44 UNEP, 2015). The notion of capacity is important to assess the integrity/degradation of the ecosystem in relation of how ecosystem services are used and managed. However, some aspects of the notion of capacity in the SEEA-EEA have not been tackled in a definitive way. Specifically: i) whether to attribute the notion of capacity to the ecosystem as a whole or to each individual ecosystem service, and ii) whether to consider ecosystem service supply independent of service demand. There is the need to address these questions because some assumptions regarding capacity are required in order to set up a complete and consistent accounting system.

Our paper investigates these two questions by applying the SEEA-EEA to the regulating ecosystem service of water purification in Europe, using in-stream nitrogen retention as a proxy for water purification. To our knowledge this is the first application of SEEA-EEA based approaches to ecosystem services measurement at a continental scale.

We begin with a brief introduction to the SEEA-EEA framework (Section 2.1), followed by the description of how the water purification ecosystem service is quantified here to be consistent with SEEA-EEA principles (Section 2.2). The results (Section 3) are expressed in terms of the SEEA-EEA procedure. The challenges raised by our case study and discussed in Section 4 aim at developing a notions of capacity able to link the accounting principles of stock and flows with ecosystem services, considering that the definition of capacity as join concept between ecology and economy is still a matter of debate.

## 2 Methods

### 2.1 Accounting for regulating ecosystems services: concepts and definitions

The SEEA-EEA framework contains ecosystem service accounts and ecosystem asset accounts for individual services and assets. As in all conventional accounting frameworks, the basic relationship is between stocks and flows. Stocks are represented by ecosystem assets. Ecosystem assets are defined as ‘spatial areas containing a combination of biotic and abiotic components and other environmental characteristics that function together’ (UN, 2014b). Ecosystem assets have a range of characteristics (such as land cover, biodiversity, soil type, altitude, slope, and so on). In accounting there are two types of flows: the first type of flow concerns changes in assets (e.g. through degradation or restoration), the second type of flow concerns the income or production arising from the use of assets. The accounting for ecosystem services regards the second type of flow although consistency is needed with the flow representing changes in ecosystem assets. According to the SEEA-EEA (UN, 2014b), the flows can be within an ecosystem asset (intra-ecosystem flow) and between ecosystem assets (inter-ecosystem flows). The combination of ecosystem characteristics, intra-ecosystem flows and inter-ecosystem flows generates ecosystem services that impact on individual and societal wellbeing.

In the SEEA-EEA tables are grouped in ecosystem assets and ecosystem services. Accounts for ecosystem assets record changes in the stocks, for example using area estimates. Accounts for ecosystem services record the flow of ecosystem services and their use by beneficiaries. Accounting for the capacity of an ecosystem to generate services is critical for determining whether the flow of an ecosystem service for human benefit is sustainable. By means of indicators describing ecosystem condition or quality, it should be possible to assess how changes in the stock of ecosystem assets affect such capacity. Indeed, the SEEA-EEA Technical Guidelines include within the ecosystem accounts an ‘ecosystem capacity account’ that should be compiled. As far as we are aware, however, there are no examples of ecosystem capacity accounts.

In order to make ecosystem capacity accounts operational, there needs to be clear definitions of key concepts and methods based on robust scientific knowledge on ecosystem functioning as well as on the relationships between ecosystem capacity, ecosystem service flows, and their benefits to humans. Edens and Hein (2013) define ecosystem services, within the context of ecosystem accounting, as the input of ecosystems to production or consumption activities. They make a strong link to economic activities by identifying the direct contribution of ecosystems to the production process. This form of accounting is feasible for provisioning services, where natural/ecological processes are combined with other kinds of capital inputs to produce goods. It is however difficult to apply to the other categories of services (cultural, regulating and maintenance). Edens and Hein (2013) acknowledge that the impact of regulating ecosystem services is external to direct economic activities or to people, stating that ‘regulating services can only be understood by analysing […] the specific mechanism through which they generate benefit’ (Edens and Hein, 2013, p.44). Our case study focusses on this point, by measuring the benefits of a regulating service – water purification.

For reporting purposes it may be necessary to aggregate ecosystem services to reduce complexity. The SEEA-EEA framework proposes three ways to aggregate ecosystem services for inclusion in accounts: i) aggregation of the various ecosystem services within a spatial area; ii) aggregation of a single ecosystem service across multiple areas within a country, and iii) aggregation of all ecosystem services across multiple areas within a country. Our case study falls within the second approach, as we account for a single ecosystem service across multiple river catchments in Europe.

### 2.2 Accounting for regulating ecosystems services: procedure

To align with SEEA-EEA definitions and methods, we use a four step procedure:

1. Identify the ecosystem service classification and the underlying conceptual framework.
2. Quantify in physical terms the targeted ecosystem service. The quantification procedure can range from simple to complex, or can be multi-tiered^4^ (Kareiva et al., 2011) because there is presently no reference framework or standard to follow.
3. Translate the quantitative assessment into monetary terms by choosing an economic valuation technique that as much consistently as possible links to the biophysical model.
4. Populate SEEA-EEA tables consistently with the resulting data.

**Fig. 1:**
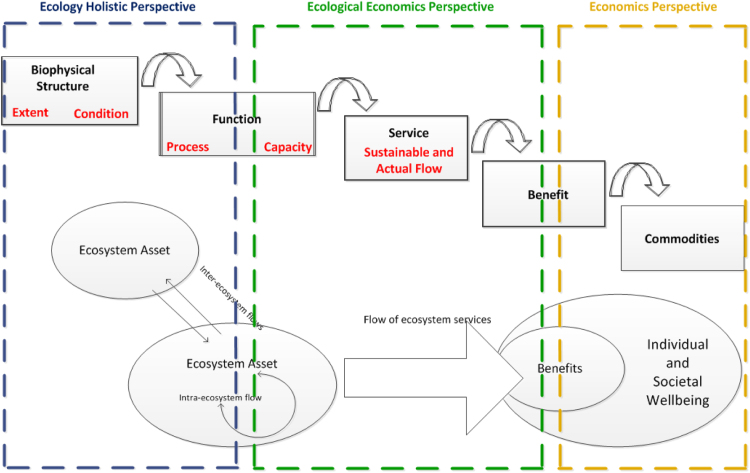
: The Ecosystem Service Cascade and the SEEA-EEA Conceptual Framework.

In step 1 we use the Common International Classification for Ecosystem Services (CICES) as proposed in the SEEA-EEA. The underlying conceptual framework is the ecosystem services cascade model (Haines-Young and Potschin, 2010). In the cascade model the biophysical structure and processes of ecosystems determine their functions, which, in turn, underpin the capacity of ecosystems to provide services. To achieve consistency between the cascade model and the SEEA-EEA framework, it is important to highlight the holistic components that guarantee the flow of individual ecosystem services and which are accounted for in the SEEA-EEA. In the cascade model's function element (Fig. 1) we distinguish a ‘process’ which occurs within the ecosystem considered as a whole, and a ‘process’ which determines the capacity of an ecosystem to generate single ecosystem services. Measurements of ecosystem functions progresses from a holistic measurement (the ecosystem as a whole) to an individual measurement (each ecosystem service). For example, processes such as nutrient and carbon cycling, as well as photosynthesis, operate within the ecosystem as a whole and depend on the condition of the ecosystem. Holistic functioning of the ecosystem and its inherent processes determines the capacity to supply single or multiple ecosystem services. In our application: the holistic process that operate within the ecosystem is nutrient cycling, the capacity is the amount of water purification that the ecosystem is able to provide now and in the future, water purification is the flow of the service provided now.

Step 2 involves the physical quantification of the selected ecosystem service. The approach most compatible with SEEA-EEA to quantify the capacity of the ecosystem to provide a service is to measure ecosystem conditions (from the ecosystem asset set of tables) using indicators such as biomass index and soil fertility. In the SEEA-EEA handbook ecosystem condition provides a link between ecosystem capacity and ability to supply ecosystem services. Here we use a biophysical model to quantify the actual flow of the ecosystem service, i.e. the amount used by society. In the supply-use accounting table the sustainable flow corresponds to the service supply, while the actual flow plus the difference between sustainable and actual flow corresponds to service use. In UNEP (2015) it is in fact left open the possibility to record what flows back to ecosystem units (i.e. the difference between sustainable and actual flow) when the supply of ecosystem service has a ‘larger scope’ (§ 4.26-F).

The actual flow of an ecosystem service is not necessarily sustainable. In overfished fisheries, for example, the actual flow exceeds the capacity of the marine ecosystem to maintain the stock, with a resulting declining stock value and the risk of collapse. A sustainable use of ecosystems requires the actual flow of the service to be equal or lower than the maximum sustainable flow that the ecosystem is able to provide. For management purposes it is therefore important to measure or estimate the sustainable flow – which remains a challenge for regulating services because it is hard to establish thresholds for sustainability.

Here we define capacity as the stock generating a sustainable flow and quantify its value by estimating the Net Present Value (NPV) of the present and future sustainable flow. We think that for accounting purposes capacity should be quantified with reference to single ecosystem services, rather than for ecosystems as a whole. In the accounting terminology, the opening stock in our approach is the capacity of the ecosystem to generate a given specific service, and it is calculated as the NPV of the ecosystem service *sustainable* flow. The changes to be recorded are the actual flow of the ecosystem service that is used by humans. The capacity is not the maximum theoretical flow the river system can generate for e.g. one year, but it represents the current and future flows measured at a sustainable rate. Capacity is thus intended as a stock (measured with NPV in money terms) and not as a flow. Consistently with these definitions, the actual flow can be higher, equal or lower than the sustainable flow, but not higher than the capacity (i.e. NPV of sustainable flows).

When the actual flow of the ecosystem service is lower than sustainable flow the implication is no degradation. Actual and sustainable flows are separate (but interconnected) tables and maps. In economic terms you might choose to only value actual flow, however the sustainable flow remains whether or not a monetary value is estimated.

If actual flow is lower than sustainable flow the capacity to provide the service remains intact. Conversely, if actual flow exceeds sustainable flow, the stock will be degraded and the capacity will be reduced. Population density, for example, affects capacity only when it drives the actual flow beyond the sustainability threshold, and its specific role can be identified provided it is explicitly included in the modelling equations behind the biophysical assessment. However, it must be acknowledged that the basis for these assumptions about capacity is that there are no other changes in the ecosystem, i.e. we assume that the condition of the ecosystem is not affected by any other changes.

Step 3 translates biophysical quantities into monetary terms. Following the SEEA-EEA guidelines, it is important to distinguish between welfare values relevant in some public policy decision making contexts, and exchange values, relevant in an accounting context. The former include consumer surplus,^5^ while the latter considers prices at which goods and services are traded and hence will include the producer surplus.^6^ One set of methodologies includes both producer and consumer surplus while the other set includes only producer surplus. Although methodologies based on exchange values might in some cases underestimate the value of ecosystem services (because they do not take into account the consumer surplus), they provide more robust values than those calculated on the basis of subjective preferences. As the focus of ecosystem accounting is on integration with standard economic accounts, ecosystem services should be estimated with reference to exchange values.

Step 4 reports the physical and monetary outputs in accounting tables in three ways:

1. Accounting for actual flow of services received by economic sectors and households;
2. Accounting for the sustainable flow of services;
3. Accounting for the capacity of ecosystems to provide a sustainable flow of the ecosystem service, calculated as the NPV of the sustainable flow.

### 2.3 Water purification accounts

The empirical objective of this case study is to value the water purification service taking place in rivers in Europe. The retention of Nitrogen (N) from point and diffuse sources is used as a proxy for water purification. Excessive nitrogen loading is a leading cause of water pollution in Europe and globally which makes nitrogen a useful indicator substance for water quality (Sutton et al., 2011, Rockström et al., 2009). We define N retention as the process of temporary or permanent removal of nitrogen taking place in the river. This includes the processes of denitrification, burial in sediments, immobilization, and transformation or simply transport (Grizzetti et al., 2015). According to this definition, N retention varies with the characteristics of the stream and of the living organisms in the aquatic ecosystem (e.g. bacteria, algae, plants), and hence depends on the ecological functioning of the system. Previous studies show that N retention is affected by N concentration in streams. Mulholland et al. (2008) showed that the efficiency of biotic uptake and denitrification declines as N concentration increases and Cardinale (2011) concluded that biodiversity in aquatic ecosystems has a positive effect on nitrogen retention. At the same time, biodiversity is threatened by high nutrient loadings in freshwater and coastal waters.

#### 2.3.1 Calculation of actual flow

We use the Geospatial Regression Equation for European Nutrient losses (GREEN) model (Grizzetti et al., 2005, Grizzetti et al., 2008, Grizzetti et al., 2012) to estimate the in-stream nitrogen retention in surface water, which is considered in this paper as the actual flow of service provision.

GREEN is a statistical model developed to estimate nitrogen (N) and phosphorus (P) flows to surface water in large river basins. The model is developed and used in European basins with different climatic and nutrient pressure conditions (Grizzetti et al., 2005) and is successfully applied to the whole Europe (Grizzetti et al., 2012, Bouraoui et al., 2009). The model contains a spatial description of nitrogen sources and physical characteristics influencing the nitrogen retention. The area of study is divided into a number of sub-catchments that are connected according to the river network structure. The sub-catchments constitute the spatial unit of analysis. In the application at European scale, a catchment database covering the entire European continent was developed based on the Arc Hydro model with an average sub-catchment size of 180 km^2^ (Bouraoui et al., 2009). For each sub-catchment the model considers the input of nutrient diffuse sources and point sources and estimates the nutrient fraction retained during the transport from land to surface water (basin retention) and the nutrient fraction retained in the river segment (river retention). In the case of nitrogen, diffuse sources include mineral fertilizers, manure applications, atmospheric deposition, crop fixation, and scattered dwellings, while point sources consist of industrial and waste water treatment discharges. In the model the nitrogen retention is computed on annual basis and includes both permanent and temporal removal. Diffuse sources are reduced both by the processes occurring in the land (crop uptake, denitrification, and soil storage), and those occurring in the aquatic system (aquatic plant and microorganism uptake, sedimentation and denitrification), while point sources are considered to reach directly the surface waters and therefore are affected only by the river retention.

For each sub-catchment i the annual nitrogen load estimated at the sub-catchment outlet (L_i_, 10^3^ kg N year^−^^1^) is expressed as following:

(1) $$L_{i}=\left(DS_{i}\times \left[1–BR_{i}\right]+PS_{i}+U_{i}\right)\times \left(1–RR_{i}\right)$$

where DS_i_ (10^3^ kg N year^−1^) is the sum of nitrogen diffuse sources in each catchment i, PS_i_ (10^3^ kg N year^−^^1^) is the sum of nitrogen point sources in each catchment i, U_i_ (10^3^ kg N year^−^^1^) is the nitrogen load received from upstream sub-catchments, and BR_i_ and RR_i_ (fraction, dimensionless) are the estimated nitrogen basin retention and river retention, respectively. In the model, BR_i i_s estimated as a function of rainfall while RR_i_ depends on the river length. For more details on model parameterisation and calibration see Grizzetti et al. (2012) and Bouraoui et al. (2009). Although simple in its structure the model GREEN is able to provide spatially distributed estimates of nitrogen river and basin retention at large scale.

The actual flow of service or in-stream nitrogen retention *N*_retained_ is simply derived from Eq. (1) as the share of nitrogen that not included in L_i_ and equals:

(2) $$N_{retained}=L_{i}\times RR_{i}\times {\left(1–RR_{i}\right)}^{-1}$$

In natural systems nitrogen retention is related to nitrogen input. The residence time of water is a key variable for in-stream nitrogen retention since it directly affects the processing time of nitrogen within an aquatic system. Longer residence times increase the proportion of nitrogen input that is retained and removed from the water. We use modelled nitrogen retention as indicator for the actual flow of the water purification service, and this assessment in turn represents the basis for the calculation of the sustainable flow (Section 2.3.2) and the translation of this assessment from physical to monetary terms (Section 2.3.3).

#### 2.3.2 Calculation of sustainable flow

Our initial hypothesis to calculate a sustainable flow of in-stream nitrogen retention is that there is a threshold in the nitrogen concentration of surface water below which the removal of nitrogen by the different ecological processes is sustainable from an ecosystem point of view. A similar threshold exists for atmospheric nitrogen deposition on terrestrial ecosystems with suggested critical nitrogen loads between 5 and 25 kg ha^−^^1^ year^−^^1^ (Bobbink et al., 2010). Here we propose to use a tentative threshold concentration of 1 mg N l^−^^1^ (Maes et al., 2012). This threshold is based on eutrophication risk. A global synthesis of published literature on the ecological and toxicological effects of inorganic nitrogen pollution in aquatic ecosystems suggests that levels of total nitrogen lower than 0.5–1.0 mg l^−1^ could prevent aquatic ecosystems from developing acidification and eutrophication (Camargo and Alonso, 2006). For potential risk of eutrophication for European surface water related to nitrogen concentration see also Grizzetti et al. (2011). This threshold concentration serves as an example for the purpose of this paper and will change depending on the vulnerability of different aquatic ecosystems to nitrogen loading. For instance, it does not apply for ecosystems naturally rich in nitrogen such as estuaries where a higher threshold could be used or for catchments with very vulnerable lakes where a lower threshold should be used. Spatially explicit sustainable targets for thresholds of total nitrogen concentration in freshwater systems can be set based on the European Water Framework Directive requirements for good or high ecological status.

Using data on average river flow (m^3^ year^−1^) in combination with the critical nitrogen concentration (1 mg l^−1^), we can calculate the critical nitrogen loading (L_crit_, 10^3^ kg N year^−^^1^) - the critical threshold below which no environmental damage is expected. Substituting the nitrogen loading L_i_ with L_crit_ in Eq. (1) and solving Eq. (2) for N_retained_ we obtain:

(3) $$N_{crit}=L_{crit}\times RR_{i}\times {\left(1–RR_{i}\right)}^{-1}$$

where N_crit_ is the critical nitrogen removal by the river network (10^3^ kg N year^−1^), assuming a critical loading L_crit_.

Next, we use the critical nitrogen load and the critical nitrogen removal function, which assumes that at or below the critical nitrogen load, the removal of nitrogen by the different ecological processes that take place in the ecosystem is sustainable and results in the optimal use of the ecosystem from an ecosystem services point of view. However, increases in nitrogen loading far above the critical loading will result in costs due to the degradation of most other ecosystem services. This hypothesis allows thus for the use of nitrogen from anthropogenic sources and the subsequent nitrogen inputs to river systems up to a level at which nitrogen concentrations reach a critical threshold. In the monetary valuation calculations nitrogen removal will be valued the most at critical nitrogen loads. The following equation is used to estimate the sustainable removal of nitrogen:

(4) $$N_{sustainable}=N_{crit}\times exp\left(–0.5\times {\left[L–L_{crit}\right]}^{2}\times {\left[1.5\times L_{crit}\right]}^{-2}\right)$$

where N_sustainable_ is the sustainable removal of nitrogen (10^3^ kg N year^−^^1^), N_crit_ is the critical removal of nitrogen (10^3^ kg N year^−^^1^), L is the nitrogen loading at the outlet of each catchment (10^3^ kg N year^−^^1^), and L_crit_ is the critical loading of nitrogen at 1 mg N l^−1^ (10^3^ kg N year^−1^).

Eq. (4) gives the sustainable in-stream nitrogen retention, also referred to in our paper as sustainable flow. It is important to stress that the exponent factor in Eq. (4) is introduced in this study to account for trade-offs that arise between water purification and other ecosystem services in conditions where nitrogen loads and concentrations are unsustainable. Studies unlike this one which analyse multiple ecosystem services delivered by aquatic ecosystems can use simply use N_crit_ as value for N_sustainable_ without applying the exponent function.

#### 2.3.3 Monetary valuation of nitrogen retention based on replacement costs

For the monetary valuation of water purification we adopt a ‘cost-based approach’. We do not use a ‘damage-based approach’ because of the difficulty to exhaustively identify all the benefits that could be lost if the water purification service offered by the ecosystem is no longer available. These benefits range from the availability of clean water for drinking or swimming, to the presence of fisheries, to the aesthetic perception that influences both recreational activities and real estate markets. The benefits from water purification also overlap in many cases with benefits from other ecosystem services, which risks to give rise to double counting. By using, instead, a cost-based approach rather than methodologies based on stated preferences we make an attempt to get closer to SEEA-EEA guidelines that preferably ask for exchange value estimates^7^; as already mentioned the choice of adopting a cost-based approach instead of a damage-based approach allows to deliver more robust and credible figures, even if it might result in an underestimation of the value of the ecosystem services.^8^ Finally, we can operationalize the underlying concept that monetary values depend upon biophysical assessments, which is a crucial prerequisite for integrated valuation.

The rationale of a cost-based approach to valuation is well known. By (partially) cleaning up discharges from human activities, aquatic ecosystems provide for free a valuable ecosystem service and thus avoid a degradation of the ecosystem that would impact on human health and living conditions. Since human activities will not stop, there will always be the need for this ecosystem service even after river bodies will not be able to provide it any longer. The operational hypothesis of our valuation exercise is that an artificial replacement would be required in order to maintain the water purification service, and replacement would entail a cost. Considering the relevant pollution sources (mainly agriculture and livestock activities together with already treated industrial and households’ discharges), the best proxy we can use as replacement cost are constructed wetlands. Wastewater treatment plants would be inappropriate because: (i) they are not applicable to the primary sector (agriculture and livestock activities) and (ii) what is discharged by the secondary sector (industrial activities) and by households is already treated by wastewater treatment plants before reaching water bodies.^9^ Constructed wetlands (CW) provide ecosystem functions similar to those delivered by aquatic ecosystems. Their construction cost refers to ecosystem engineering work, which is more objective than values obtained through stated preferences, with a survey questioning citizens on the value they would place on nitrogen retention. The rationale is that artificial wetlands are also able to retain N present in relatively low concentrations, as opposed to urban wastewater treatment plants that need high concentration of the pollutant for efficient removal. A review of the value attributed to nitrogen retention is available from a previous study (La Notte et al., 2012a) where it is clearly shown how the choice of replacement costs is very popular among environmental economists. Wastewater treatment plants are much more expensive than CW; moreover, in our valuation exercise (following subsection) we differentiate between typologies of CW in order not to overestimate the cost, in fact the more extensive typology of CW (Free Water Surface) is the less expensive solution. We thus use the cost of CWs as proxy for the valuation of nitrogen retention, which represents a proxy for water purification. Specifically, the amount of nitrogen that is retained and removed by rivers and lakes will be converted to a CW area equivalent, i.e. the total area (ha) of CW that is needed to result in the same nitrogen retention as the river network in each sub-catchment. Once we have this CW area equivalent, we calculate the costs of the corresponding typology of CWs based on cost data.

Differently from previous applications undertaken on water purification (La Notte et al., 2015, La Notte et al., 2012a) the monetary values here are not derived from other studies but calculated *ad hoc* for the specific engineering works hypothesized.

The typologies of CW are differentiated according to the types of pollutant sources (Kadlec and Wallace, 2009).

Free Water Surface (FWS) CWs are densely vegetated basins that contain open water, floating vegetation and emergent plants. They basically need soil to support the emergent vegetation. The FW constructed wetlands reproduce closely the processes of natural wetlands, attracting a wide variety of wildlife, namely insects, mollusks, fish, amphibians, reptiles, birds and mammals (Kadlec and Wallace, 2009). FWS-CWs are the best choice for the treatment of nutrients from diffuse primary sector activities.

Horizontal subsurface Flow (HF) CWs consist of waterproofed beds planted with wetland vegetation (generally common reeds) and filled with gravel. The wastewater is fed by a simple inlet device and flows slowly in and around the root and rhizomes of the plant and through the porous medium under the surface of the bed in a more or less horizontal path until it reaches the outlet zone. HF-CWs represent the best choice for treating point sources.

Kadlec and Knight (1996)’s method for the sizing of CWs systems describes nitrogen removal with first-order plug-flow kinetics:

(5) $$ln\left(\frac{c_{e}-c^{*}}{c_{i}-c^{*}}\right)=\frac{-k}{q}\;q=\frac{365\cdot Q}{A_{s}}$$

(6) $$A_{s}=\frac{365\cdot Q}{k}ln\left(\frac{c_{i}-c^{*}}{c_{e}-c^{*}}\right)$$

where:

• A_s_: surface of the CWs (m^2^)
• c_e_: outlet concentration (mg l^−^^1^)
• c_i_: inlet concentration (mg l^−1^)
• c*: background concentrations, for nitrate assumed at 0 mg l^−1^
• k: areal constant of first order (m year^−1^); for nitrogen removal k is temperature dependent: K˭K_20_∙θ^(T-20)^ (K_20_ takes values of 41.8 for HF and 30.6 for FWS; θ takes values 1.102 for HF and FWS, T is the temperature of the water in degree Celsius)
• Q: hydraulic load (m year^−^^1^)
• q: mean flow (m^3^ day^−1^)

The flow Q is separated in two different sub-flows: a first one containing only nitrogen from diffuse sources, which is calculated as the product of surface basin and annual precipitation (supposing a completely impervious basin); and a second one containing only nitrogen from point sources, whereby the point input sources (kg) were converted according to Eq. (6) to a flow value (m^3^ day^−1^) by using population data and by assuming person equivalents (a person equivalent corresponds to 12 g N day^-1^ and discharges 250 l drinking water per day).

We assumed that the nitrogen load removed by HF and FWS is proportional to the ratio between non-point and point sources discharging into the basin. In order to assess the ratio between c_i_ and c_e_ (Eq. (5)) we perform the calculations in Eqs. (7), (8).

For diffuse sources:

(7) $$\frac{C_{i}}{c_{e}}=\frac{(L_{i}+({\mathrm{DS}}_{i}\times \left(1-{\mathrm{BR}}_{i}\right))}{L_{i}+\left({\mathrm{DS}}_{i}\times \left(1-{\mathrm{BR}}_{i}\right)\right)-({\%N}_{\mathrm{FWS}}\times \mathrm{NR})}$$

where:

L_i_: Load at catchment inlet (10^3^ kg year^−1^)
DS_i_: Diffuse sources at catchment (10^3^ kg year^−1^)
BR_i_: Basin retention (dimensionless)
% N _FWS_: 1 - Percentage of point sources
NR: In-stream nitrogen retention (10^3^ kg year^−1^)
  For point sources:
  (8) $$\frac{C_{i}}{c_{e}}=\frac{(L_{i}+{\mathrm{PS}}_{i})}{L_{i}+{\mathrm{PS}}_{i}-({\%N}_{\mathrm{HW}}*\mathrm{NR})}$$
  where:
  L_i_=Load at catchment inlet (10^3^ kg year^−1^)
  PS_i_=Point input sources to the river at catchment (10^3^ kg year^−1^)
  % N_HF_=Percentage of point sources
  NR: In-stream nitrogen retention (10^3^ kg year^−1^)

Once we have the CW area equivalent, we can calculate the costs of the corresponding typology of CWs. Total costs include direct construction costs, indirect construction costs and costs of labour and material.

To include economies of scale in construction costs, we implement the relationship between surface and construction costs presented by Kadlec and Wallace (2009), with a factor of 0.77 for the conversion US dollar to euro.^10^

(9) $$FWS\;Cost\;\left(€\right)=.77\times 194\times A^{0.690}$$

where A stands for area in ha and 0.03 ha<A<10000 ha; and

(10) $$HF\;Cost\;\left(€\right)=0.77\times 652\times A^{0.704}$$

where A stands for area in ha and 0.005 ha<A<20 ha.

Indirect costs (not including the cost of land acquisition^11^) have been included as a standard percentage (30%) of construction costs.^12^

Labour cost values have been extracted from the Eurostat labour statistics, which reports costs from 1997 to 2009. For countries with missing data, we estimate approximate values based on those of adjacent countries with similar economic conditions. The costs of filling materials are obtained by a direct survey conducted among CW designers and builders in different European countries and by data available in the peer-reviewed literature.

To account for price differentials across countries, construction costs have been divided in three components: (1) a fixed component (including waterproofing, excavation, plants, concrete elements, piping, etc.); (2) labour costs; (3) filling materials costs.

For each country the total cost (€ m^−2^) is obtained as the sum of fixed costs, labour costs and filling material cost for HF and as sum of fixed costs and labour cost for FWS. On the ground of a series of case studies examined, we assume an operating and maintenance (O&M) cost equal to 3850 € ha^-1^ for FWS and 7700 € ha^−^^1^ for HF.

The building value that we calculate refers to the whole building project. What we need in our valuation is an annual flow, we thus need to calculate it. For the estimation of the annual flow from the total building costs, we can use the standard equation:

(11) $$a=\frac{Y*i*{(1+i)}^{\mathrm{LE}}}{{\left(1+1\right)}^{\mathrm{LE}}-1}$$

where:

a: yearly amount of building costs (euro)
Y: total building costs
i: discount rate (in our application set at 3%^13^)
LE: Life Expectancy of the CW (20 years).

We should take into account on one hand the economy of scale effect, and on the other hand the fact that different countries in Europe have different costs. The two aspects cannot be calculated together because the imposition of fake thresholds would unrealistically affect the final result. We thus calculate separately the economy of scale effect and the price difference effect. After few simulations were run (La Notte et al., 2012b), the most reliable outcomes result from the combination that considers a 70-30 breakdown, i.e. 70% of the cost is based on an assessment of the price difference effect and 30% of the cost is based on the economies of scale model (Eqs. (9), (10)).^14^

## 3 Results

We present the accounts at two spatial scales: i) at the European scale, to show how service capacity and service flow can be quantified through the accounting tables proposed by the SEEA-EEA, and; ii) at the country scale, to put sustainable and actual flow and valuation estimates into context. We report monetary estimates in constant year 2000 values: valuation is used here as a translation in monetary terms of the biophysical assessment, and including inflation in the estimates would overshadow their comparability over time. Using current rather than constant prices is obviously feasible and may be desirable for different purposes.

In Europe, over the 20-year time period considered (1985–2005), total nitrogen input to river basins varies between 50 and 80 million ton, the largest share originating from the agricultural sector and entering the basin as diffuse sources. This total represents the combined input of different nitrogen sources on the land after take up by crops. After basin retention (i.e. the nitrogen that is retained in soils and groundwater), around 5 million tons reach the river network. Nitrogen emissions from industries and households enter the river network as point sources and amount to 1.1 million ton of nitrogen.^15^

Table 1, Table 2 present stock (capacity) and flow accounts, respectively, of the delivery of water purification services by the European river network as indicated by in-stream nitrogen retention. We calculate that replacing this ecosystem service capacity would require approximately one million ha of constructed wetland, representing a net present value of between 310 billion € in 1990 and 459 billion € for the year 2005 (Table 1).

The flows of total annual service vary between 21 and 31 billion euro assuming sustainable service delivery. The actual service flow aggregated at the European scale is worth around 16 billion euro annually (Table 2). Economic sectors and households are the polluting subjects who actually use the water purification service. The total values aggregated for Europe suggest that the sustainable flow is higher than the actual flow. Relative values disaggregated at the country level will read differently.

The separation between the primary sector and other economic activities and households has been determined by the features of the biophysical model that explicitly differentiate retention values for diffuse source (that are indeed mostly due to agriculture activities) and point sources. The possibility to frame the results according to economic sectors (by using of course the same classification) offers the possibility to integrate this information with economic accounts, all expressed in monetary terms.

**Fig. 2:**
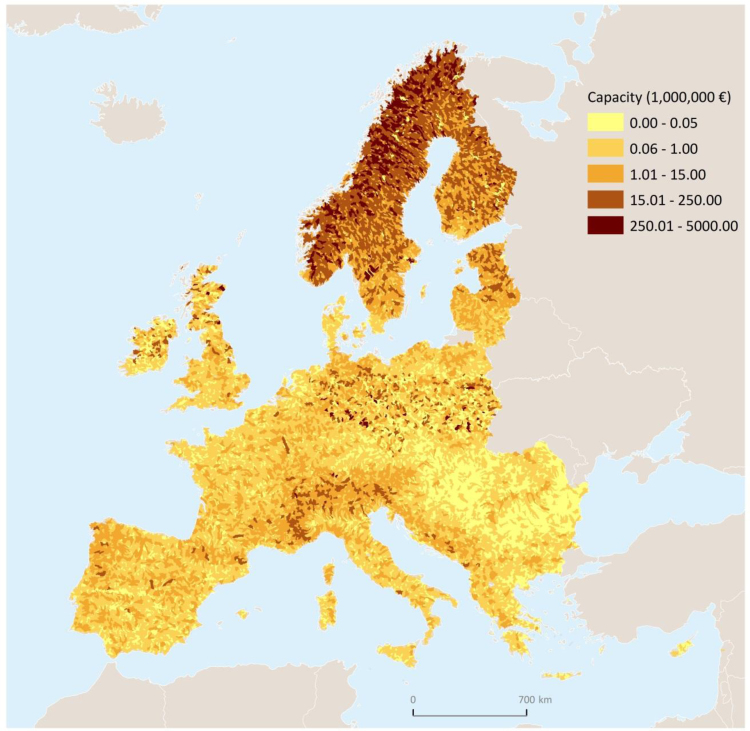
Map of the capacity to deliver sustainable nitrogen retention (euro).

**Fig. 3:**
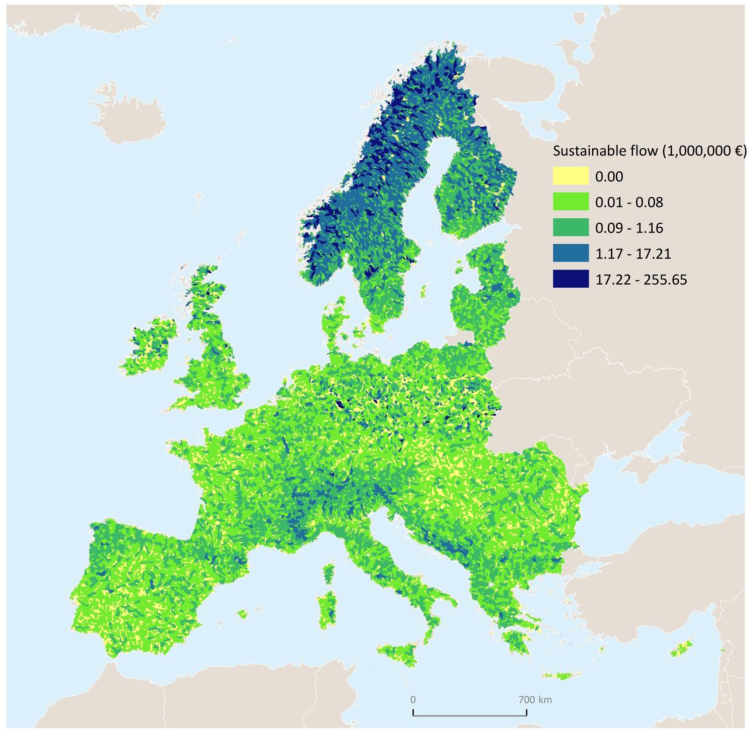
Map of sustainable nitrogen retention or sustainable flow (euro).

**Fig. 4:**
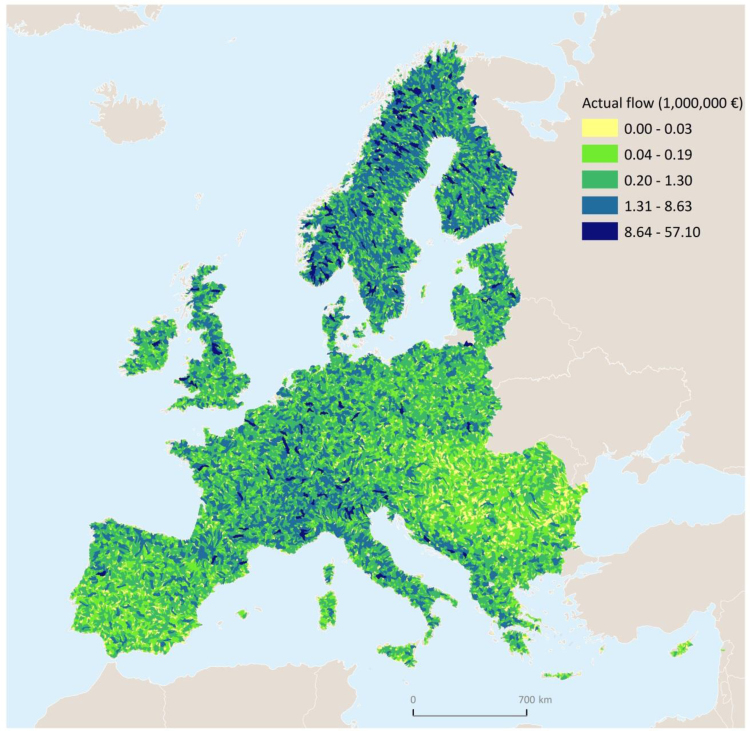
Map of actual nitrogen retention or actual flow (euro).

In Table 3, Table 4 we report estimates expressed respectively in 10^3^ kg km^−1^ year^−1^and euro km^−1^ year^−^^1^, so as to assess sustainability independently of the size of the country. Total values are mapped in Fig. 2, Fig. 3, Fig. 4, at the European level as well as for the 34 countries covered by the model extent.

Table 3, Table 4 account for the ecosystem service flow at a country level, estimated in physical and monetary terms, respectively, for 1985, 1995 and 2005. Table 3 also presents statistics on the total size of river basins and the national river network as well as total nitrogen emissions. These latter statistics can be used to convert the accounts expressed per kilometre into national totals. The results reported in Table 3 demonstrate that for many countries the sustainable flow, measured in physical units, is below the actual flow. Consequently, monetary values based on physical accounts show their same pattern (Table 4). Please be aware that sustainable flow does not represent the whole possible flow. It does represent the level of the flow that can be used without degrading the capacity of the ecosystem to provide it. Actual flow can indeed be higher than the sustainable flow but this over-exploitation will affect the degradation of the ecosystem and thus future levels of sustainable flow.

Furthermore, Table 3 shows that in most countries total nitrogen emissions have gradually declined between 1985 and 2005 (see Bouraoui et al., 2011 and Grizzetti et al., 2012 for an analysis of changes in nutrient water emissions in Europe in the last decades). Given the positive relation between nitrogen input and actual in-stream nitrogen retention, the physical flow accounts follow this emission trend and show, on average, a decline in the average amount of nitrogen retained per unit length of river network. How far a country is from a sustainable situation depends on the magnitude of past N inputs. Consider the Netherlands (Table 3): they have substantially decreased N input in the last 15 years, but the difference between actual N emissions and the sustainable limit is nonetheless the largest in Europe. For almost all countries the actual flow is higher than the sustainable flow, which means that river ecosystems in Europe are progressively degrading as a result of nitrogen pressure. Sustainable use is achieved in Estonia, Finland, Norway and Sweden, where actual flows for 2005 were on average lower than the sustainable flows. In all other countries considered, in-stream nitrogen retention occurs at unsustainable levels.

The apparently contrasting results between Table 1, Table 2, Table 3, Table 4 offer few lines of thought. Considering absolute values (sum of the total) provide a rather different picture than relative values (average per km); it is thus important to establish what is the figure we choose to analyse and for what purpose. Moreover, the countries to be included does affect the final value: including or omitting one or few countries can overturn the results, if these countries have economic activities with a highly impacting effect and/or a considerable size.

A few important points are worth highlighting. (i) The capacity to generate sustainable nitrogen retention, shown in Fig. 2, and the sustainable flow (Fig. 3) exhibit the same distribution, but with a different order of magnitude.^16^ Whereas (ii) both distribution and order of magnitude differ in the trend of actual flows (Fig. 4) relative to capacity. (iii) Sustainable flow (Fig. 3) and actual flow (Fig. 4) exhibit different distributions but same order of magnitude.

Interesting observations emerge also from the monetary flow accounts (Table 4). Firstly, variation between countries is much higher than observed in the physical accounts. This is largely the result of different price levels among different countries in Europe, with highest values for Scandinavian countries and lowest values for Balkan countries. Secondly, the annual variation in actual flow within countries is limited as a result of the high fixed costs relative to variable costs used in the replacement cost model. These points will be discussed more in depth in the Discussion.

The accounts reported in Table 1 (capacity intended as a stock measure) should always be consistent with those reported in Table 2 (flows). Consistency is guaranteed by the use of the same biophysical model to which, in the case of the assessment of sustainable flows, a critical threshold concentration is applied.

Finally, it should be recalled that the nitrogen retention takes place in soils, surface water including streams, river and lakes, wetlands, and coastal and marine sediments. Our accounts, however, are limited to the river network.

## 4 Discussion

The crucial note we address with this case study is the definition, in accounting terms, of stocks and flows of ecosystem services. Ecosystem services depend on the functioning and health of the ecosystem as a whole. Ecosystem resilience is related to the capacity of ecosystems to generate ecosystem services, now and in the future. However, the notion of capacity is controversial. In Burkhard et al. (2014) a difference is made between ‘ecosystem service potential’ (used as synonym of capacity), defined as the hypothetical maximum yield of selected ecosystem services, and ‘ecosystem service flow’, defined as the used set of ecosystem services. This definition of ecosystems services potential (or capacity) follows the notion of stock, as long as it is clear that ‘ecosystem service potential’ differs from ‘ecosystem service potential supply’. Potential supply *versus* actual flow is what we define as sustainable flow *versus* actual flow. In Villamagna et al. (2013) service capacity is the ‘ecosystem's potential to deliver services based on biophysical properties, social condition, and ecological functions’ (p. 116). This definition theoretically links ecosystem services to the notion of stock. However, in both Villamagna et al. (2013) and Schröter et al. (2014), examples are provided in which the flow of ecosystem service can be higher than the capacity. In our approach we suggest that accounting notion of capacity should be defined as the stock generating the sustainable flow. Thus, the actual flow can be higher than the sustainable flow, but the result is a depletion of the ecosystem's capacity to generate future flows.

Our application identifies several challenges that need to be addressed before a standard framework for integrated ecosystem and economic accounting can be proposed. The first is the difference between potential flows and sustainable flows. Potential flow is the maximum flow of a given service that the ecosystem is able to generate; sustainable flow is the flow that does not exceed the regeneration rate. For provisioning services it is possible to quantify the difference between the two. For regulating and maintenance services it is possible to measure the sustainable flow once a sustainability threshold has been identified, but it is unclear whether it would be possible to measure potential flow. This is a key point that needs to be addressed in order to make the accounting for ecosystem services operational and rigorous. Even establishing a sustainability threshold is not trivial because the conditions and vulnerability of ecosystems vary in space and time.

One feature of our application that needs to be highlighted is the use of constructed wetlands for valuing the NPV of water purification sustainable flow. Ideally the quantification of ecosystem capacity (in our application: the NPV of the sustainable flow) and services (actual and sustainable flows) should be based on the assessment undertaken in physical terms and not be dependent on the valuation methodology. In many cases, however, this turns out to be not possible. In our case study, for example, the available biophysical model (GREEN) is based on a statistical approach, using regression analysis to build a statistical relation between retention and explanatory variables such as land cover, climate, and so on. The model does not include equations representing the physical functions of the ecosystem. For future applications and wherever possible, process-based models should be used to quantify stock-capacity and flow-service.^17^

Directly related with the choice of using CWs as replacement cost is the choice of lifetime of the resource and of the discount rate used in calculating the Net Present Value (i.e. the lifetime of the resource in terms of number of years depends on constructed wetlands as an engineering work rather than on water purification as an ecosystem service). Moreover, we not only consider operation and maintenance costs (that are annual costs) but we also 'incorporate' building costs considered over the 20 years of the CW life. One important consequence is that fixed costs play the most important role, consistently with our hypothesis that substitute costs (building and annual maintenance of artificial capital) have to be incurred once natural processes have been impaired. Underlying assumptions must however be kept in mind when comparing monetary values resulting from different valuation techniques. Finally, although CW are likely to affect the concentration of other pollutants and to provide other ecosystem services, we only related CW to nitrogen emissions, the pollutant we used as proxy of anthropic pressure on the water purification service. In a future application the application of the whole cost should be maybe re-proportionated based on feasible hypotheses.

Another point highlighted by our application is the critical role played by the way in which the sustainability threshold is calculated and spatially differentiated according to physical conditions. The latter, in fact, causes the sustainable flow be very sensitive to changes in emissions. A sensitivity analysis (available as Supplementary Material) has been conducted and we demonstrate that the drivers of changes in the final outcome mainly depend on the biophysical assessment: 56% depends on the model input and parameters, 27% depends on the parameters used to size the area of CW necessary to retain the amount of N and only 17% depends on the purely economic figures, i.e. building and O&M costs and their coefficients, the discount rate and life expectancy of the CWs.

As a final note, the exercise presented in this paper shows once more that quantifying and valuing ecosystem services often involves complex, fit for purpose models. This imposes heavy reliance on derived data. If developing data standards is generally considered one of the objectives of ecosystem service accounting, we therefore suggest that a standard on metadata settings (e.g. Crossman et al., 2013), in addition to a standard for data, is equally required.

## 5 Conclusions

Ecosystem service accounting is in its infancy and substantial work is needed to refine and test methods for use in National Accounts. Our case study addresses the issue of capacity in integrated ecosystem and economic accounting by structuring ecosystem services within a consistent stocks and flows perspective – something not fully addressed in previous applications of ecosystem services accounting.

The first key message from our application is that capacity can be calculated for ecosystem services one-by-one. The second key message is that capacity can be intended as the stock that provides the sustainable flow of the service. The third key message is that the sustainable flow must be calculated jointly with the actual flow. Current use of any ecosystem service should be assessed against sustainable use. The capacity should describe the sustainable flow of each service that the ecosystem can generate, even if not currently fully exploited. The common underlying element underneath the first two messages is that the physical flow accounts provide the basis upon which other accounting tables are built.

By calculating capacity and flow, in our case study, we demonstrate how to make the theory and concepts described in the SEEA-EEA operational for use in upcoming standards of integrated ecosystem and economic accounting.

## Figures and Tables

**Table 1:** Total capacity of the European river network to generate in-river nitrogen retention services at sustainable level expressed in physical terms and in monetary terms (constant price 2000). Data based on an assessment for 34 European countries.^a^

Physical terms	Required area of constructed wetlands (10^3^ ha)
1985	862.78
1990	803.90
1995	842.63
2000	895.82
2005	1 174.51
Monetary terms	Net present value of the stock (billion €)
1985	335.78
1990	310.72
1995	327.67
2000	345.09
2005	458.86

a Capacity is calculated as NPV of the sustainable flow. Actual flow is thus here not considered.

**Table 2:** Total flow of in-river nitrogen retention to the economy and society, in monetary terms (constant price 2000, billion € year^−^^1^). Data based on an assessment for 34 European countries.

	Agriculture, forestry and fishing	Other economic activities and households
Sustainable flow		
1985	22.461	0.109
1990	20.781	0.105
1995	21.920	0.105
2000	23.084	0.111
2005	30.736	0.107
Actual flow		
1985	16.023	0.181
1990	16.010	0.176
1995	16.059	0.162
2000	15.979	0.176
2005	15.879	0.168

**Table 3:** Physical flow for in-stream nitrogen retention at national scale.

**Country**	**River area covered by the study (km**^**2**^**)**	**River network (km)**	**Total nitrogen input (ton km**^**−2**^**year**^**−1**^**)**			**Sustainable river nitrogen removal (10**^**3**^**kg km**^**−1**^**year**^**−1**^**)**			**Actual river nitrogen removal (10**^**3**^**kg km**^**−1**^**year**^**−1**^**)**		
			1985	1995	2005	1985	1995	2005	1985	1995	2005
Albania	27061	2265	5.89	5.22	5.50	0.46	0.39	0.30	2.96	3.27	3.52
Andorra	547	39	5.81	7.36	7.94	0.23	0.15	0.06	0.70	0.88	1.17
Austria	83943	7456	8.15	7.05	6.61	0.47	0.51	0.64	3.25	3.06	2.59
Belgium	29201	2540	20.97	18.67	18.15	0.03	0.01	0.06	5.07	5.96	4.88
Bosnia and Herzegovina	50523	4413	5.61	3.84	3.65	0.44	0.50	0.52	2.11	1.94	1.89
Bulgaria	110246	9681	20.39	5.92	5.11	0.63	0.85	0.76	5.70	5.15	5.57
Croatia	49611	4894	9.87	6.57	5.50	0.55	0.75	0.91	5.19	4.43	3.91
Cyprus	5617	452	7.00	20.08	18.37	0.00	0.00	0.00	0.16	0.14	0.18
Czech Republic	79430	7077	16.51	10.28	10.37	0.55	0.56	0.56	3.95	2.76	2.57
Denmark	27423	1968	19.68	18.95	16.72	0.00	0.00	0.00	2.02	1.39	1.60
Estonia	39531	3507	6.03	4.40	5.34	0.25	0.37	0.43	1.07	0.66	0.29
Finland	315292	26662	2.19	2.22	2.71	0.65	0.65	0.65	0.37	0.35	0.30
France	530446	48075	11.04	10.32	10.62	0.74	0.52	0.81	4.38	6.02	3.87
Germany	349749	32361	18.39	13.19	12.25	0.73	0.66	1.15	8.65	8.10	5.32
Greece	92992	7416	16.18	9.00	6.88	0.11	0.05	0.04	1.60	1.93	1.97
Hungary	92541	9726	15.96	9.90	9.52	0.11	0.31	0.39	5.80	4.61	4.36
Ireland	55233	4260	13.57	16.56	14.31	0.36	0.36	0.36	2.25	2.60	2.10
Italy	268665	24492	11.18	10.02	9.38	0.76	0.71	0.85	6.71	6.99	6.37
Latvia	61803	5954	6.25	4.06	3.60	0.98	1.31	1.38	3.72	2.36	2.08
Lithuania	65325	6421	9.46	5.44	5.74	0.12	0.56	0.51	5.14	3.19	3.32
Luxembourg	3614	381	15.45	13.31	13.05	0.01	0.00	0.05	4.19	4.92	3.25
Macedonia	24448	1847	8.26	5.49	4.32	0.09	0.04	0.04	2.23	2.62	2.60
Netherlands	29789	2871	36.36	31.86	27.59	0.57	0.59	1.63	13.29	13.32	8.93
Norway	238212	19440	1.28	1.46	1.65	0.66	0.66	0.65	0.28	0.29	0.24
Poland	308161	28062	11.68	8.50	7.77	0.59	0.75	0.79	9.25	6.07	5.72
Portugal	82457	7453	4.80	5.02	9.34	0.26	0.21	0.42	2.41	2.55	1.91
Romania	239315	23531	13.24	7.30	5.84	0.66	0.94	0.80	6.09	5.29	5.79
Serbia incl. Montenegro	100400	8864	10.70	6.88	6.68	0.45	0.69	0.70	6.23	5.37	5.50
Slovakia	49149	4316	10.63	7.75	6.07	0.04	0.11	0.13	1.54	1.08	1.03
Slovenia	20443	1880	10.84	7.46	6.63	0.46	0.55	0.59	1.74	1.38	1.26
Spain	479392	42510	18.97	37.87	16.55	0.24	0.24	0.18	1.36	1.39	1.69
Sweden	425284	38678	2.14	2.19	2.37	0.79	0.80	0.79	0.54	0.46	0.36
Switzerland	42202	3559	10.33	9.08	7.30	2.24	2.02	2.54	5.36	6.09	4.20
United Kingdom	197677	16262	15.82	15.72	13.51	0.27	0.27	0.27	2.76	2.50	2.37

**Table 4:** Monetary flow (constant price 2000) for in-stream nitrogen retention at national scale.

**Country**	**Sustainable river nitrogen removal (euro km**^−^^**1**^**year**^−^^**1**^**)**			**Actual river nitrogen removal (euro km**^**−1**^**year**^**−1**^**)**		
	**1985**	**1995**	**2005**	**1985**	**1995**	**2005**
Albania	12,752	9,683	7,332	35,138	34,634	35,361
Andorra	10,055	5,250	1,776	31,015	30,907	30,614
Austria	12,657	12,856	14,640	20,542	20,612	20,649
Belgium	318	64	478	44,390	42,487	41,798
Bosnia and Herzegovina	7,786	8,694	8,052	24,444	24,284	24,376
Bulgaria	1,923	1,130	774	11,297	11,646	11,828
Croatia	1,822	2,378	2,940	16,227	16,175	16,144
Cyprus	277	662	407	6,149	6,131	6,001
Czech Republic	14,161	19,435	21,862	19,276	19,009	19,181
Denmark	0.09	3.05	52.88	60,580	60,218	60,201
Estonia	32,738	11,421	45,349	40,097	40,039	39,715
Finland	178,935	156,191	206,894	74,929	74,920	74,619
France	7,628	5,139	7,592	44,389	44,816	44,063
Germany	20,356	21,418	33,449	32,196	32,756	31,659
Greece	1,044	708	746	26,127	25,769	25,492
Hungary	80	155	171	5,212	5,164	5,163
Ireland	22,948	20,276	25,136	40,970	41,083	40,894
Italy	8,581	8,211	9,964	45,253	45,609	45,251
Latvia	3,404	13,232	22,569	39,898	39,416	39,325
Lithuania	143	3,119	3,809	32,564	31,950	32,372
Luxembourg	204	46	1,114	50,247	50,661	48,853
Macedonia	2,881	1,436	1,453	31,662	32,412	32,038
Netherlands	58	69	253	34,595	34,176	33,725
Norway	337,364	310,686	408,656	86,814	86,895	86,795
Poland	21,714	23,299	25,525	25,478	25,144	25,232
Portugal	2,728	2,538	8,197	17,513	17,219	16,600
Romania	578	930	399	5,709	5,673	5,761
Serbia incl. Montenegro	3,874	3,131	1,398	13,043	13,079	13,141
Slovakia	319	1,187	1,228	7,913	7,777	7,759
Slovenia	7,519	11,639	12,029	25,749	25,399	25,047
Spain	4,570	4,649	3,863	19,539	19,288	17,622
Sweden	208,661	223,885	344,206	79,913	80,159	80,073
Switzerland	56,889	51,682	77,340	88,437	89,299	87,967
United Kingdom	19,069	20,707	20,638	45,209	44,887	44,685

## Footnotes

1 See for example *Wealth Accounting and Valuation of Ecosystem Services* (http://www.wavespartnership.org/waves/).

2 The first release was published in 1953. Revisions took place in 1968, 1993 and 2008.

3 Depletion of natural assets recorded in SNA refers only to those natural assets that constitute economic goods. However, SNA does include some natural resources such as energy resources. For those resources the SNA include a measure of depletion in the balance sheets, but not in production or income accounts.

4 An example is provided by the MAES (Mapping and Assessment of Ecosystem Services) initiative. In MAES (2012) Tier 1 requires mapping ecosystem services by using available indicators; Tier 2 requires mapping ecosystem services by linking different indicators with land use data; Tier 3 requires mapping ecosystem services through model-based approaches.

5 Consumer surplus is the gain obtained by consumers because they are able to purchase a product at a market price that is less than the highest price they would be willing to pay.

6 Producer surplus is the amount that producers benefit by selling at a market price that is higher than the least that they would be willing to sell for, which is related to their production costs.

7 It must be acknowledged that the question of which valuation approaches are appropriate for the estimation of exchange values used in accounting is still a matter of discussion.

8 As already explained cost-based approach only consider producer surplus.

9 In the model GREEN the discharges from wastewater treatment plants are treated as point sources.

10 The 0,77 was the exchange rate when our study was completed.

11 The cost of land acquisition is not considered here because of the difficulties of having an available an exhaustive database about the cost of land according to its uses for all EU member states. In La Notte et al. (2012b) an attempt is made to estimate how higher would be the value of water purification if the values include the costs of land acquisition. The results suggest that the range of values to account for could even double the initial value; building and operation costs of CWS only represent a minimum value to be considered when assessing the value of water purification. However, a more exhaustive and accurate study needs to be undertaken in order to build a reliable relationship should be established between the typology of land and the rents it generates.

12 Indirect costs usually include: Engineering and permitting activities, non-construction contractor costs, construction observation and start-up services and contingency and escalation: escalation is an allowance for inflation. Contingency is a percentage of the base cost to cover error in human judgment. Contingency allotments of 10–30% are typically used. The hypothesis of 30% is based on field experience reported by engineering companies that build CWs.

13 The discount rate has been chosen according to the SEEA-CF guidelines reported in Annex A5.2 (UN, 2014a).

14 A complete description of the cost-based approach is available in La Notte et al. (2012b). A sensitivity analysis related to the model GREEN outcomes is reported in Grizzetti et al. (2012).

15 All estimates related to nitrogen input can be retrieved from Grizzetti et al. (2012).

16 The two maps are built from the same data set: in the case of the sustainable flow we map the amount per year (the flow), in the case of capacity we map the amount as NPV over 20 years (the stock). This explains the difference in terms of order of magnitude and the similarity in terms of spatial distribution.

17 For a discussion of different modelling approaches to estimate water nitrogen retention see Grizzetti et al. (2015).

## References

[bib1] Bartelmus P., Stahmer C., van Tongeren J. (1991). Integrated environmental and economic accounting: framework for a SNA satellite system. Rev. Income Wealth.

[bib2] Bobbink R., Hicks K., Galloway J., Spranger T., Alkemade R., Ashmore M., Bustamante M., Cinderby S., Davidson E., Dentener F., Emmett B., Erisman J.-W., Fenn M., Gilliam F., Nordin A., Pardo L., De Vries W. (2010). Global assessment of nitrogen deposition effects on terrestrial plant diversity: a synthesis. Ecol. Appl..

[bib3] Bouraoui F., Grizzetti B. (2011). Long term change of nutrient concentrations of rivers discharging in European seas. Sci. Total Environ..

[bib4] Bouraoui, F., Grizzetti, B., Aloe, A., 2009. Nutrient discharge from rivers to seas for year 2000. Joint Research Centre Scientific and Technical Research Series EUR 24002 EN. Luxembourg: Publications Office of the European Union, doi: 10.2788/38971.

[bib5] Burkhard B., Kandziora M., Hou Y., Müller F. (2014). Ecosystem service potentials, flows and demand – concepts for spatial localisation, indication and quantification. Landsc. Online.

[bib6] Busch M., La Notte A., Laporte V., Erhard M. (2012). Potentials of quantitative and qualitative approaches to assessing ecosystem services. Ecol. Indic..

[bib7] Camargo J.A., Alonso A. (2006). Ecological and toxicological effects of inorganic nitrogen pollution in aquatic ecosystems. A global assessment. Environ. Int..

[bib8] Cardinale B.J. (2011). Biodiversity improves water quality through niche partitioning. Nature.

[bib9] Crossman N.D., Burkhard B., Nedkov S., Willemen L., Petz K., Palomo I., Martín-Lopez B., Boyanova K., Alkemade R., Egoh B., Drakou E.G., Dunbar M., Maes J. (2013). A blueprint for mapping and modelling ecosystem services. Ecosyst. Serv..

[bib10] Daily G.C., Polasky S., Goldstein J., Kareiva P.M., Mooney H.A., Pejchar L., Ricketts T.H. (2009). Ecosystem services in decision making: time to deliver. Front. Ecol. Environ..

[bib11] Díaz S., Demissew S., Carabias J. (2015). The IPBES conceptual framework — connecting nature and people. Curr. Opin. Environ. Sustain..

[bib12] Edens B., Hein L. (2013). Towards a consistent approach for ecosystem accounting. Ecol. Econ..

[bib13] European Commission, 2011b. Our life insurance, our natural capital: an EU biodiversity strategy to 2020. Communication from the Commission to the European Parliament, the Council, the Economic and Social Committee and the Committee of the Regions. COM (2011) 244 Final, Brussels.

[bib14] European Commission, 2011a. Communication from the commission to the european parliament, the council, the european economic and social committee and the committee of the regions: Roadmap to a Resource Efficient Europe. COM (2011) 571 Final, Brussels, 20.9.2011.

[bib15] European Environmental Agency, 2006. Land accounts for Europe 1990–2000. Towards integrated land and ecosystem accounting, Office for Official Publications of the European Communities: Luxembourg, ISBN 92-9167-888-0.

[bib16] European Environmental Agency, 2010. Ecosystem accounting and the cost of biodiversity losses. The Case of Coastal Mediterranean Wetlands. EEA Technical Report No. 3/2010, Office for Official Publications of the European Union: Luxembourg, ISBN 978-92-9213-092-3.

[bib17] European Environmental Agency, 2011. An Experimental Framework for Ecosystem Capital Accounting in Europe. EEA Technical Report No. 13/2011, Publications Office of the European Union: Luxembourg, ISBN 978-92-9213-233-0.

[bib18] Grizzetti B., Bouraoui F., de Marsily G., Bidoglio G. (2005). A statistical method for source apportionment of riverine nitrogen loads. J. Hydrol..

[bib19] Grizzetti B., Bouraoui F., De Marsily G. (2008). Assessing nitrogen pressures on European surface water. Glob. Biogeochem. Cycles.

[bib20] Grizzetti B., Bouraoui F., Billen G., van Grinsven H., Cardoso A.C., Thieu V., Garnier J., Curtis C., Howarth R., Jones P., Sutton M., Britton C., Erisman J.W., Billen G., Bleeker A., Greenfelt P., van Grinsven H., Grizzetti B. (2011). Nitrogen as a threat to European water quality. In The European Nitrogen Assessment.

[bib21] Grizzetti B., Bouraoui F., Aloe A. (2012). Changes of nitrogen and phosphorus loads to European seas. Glob. Change Biol..

[bib22] Grizzetti B., Passy P., Billen G., Bouraoui F., Garnier J., Lassaletta L. (2015). The role of water nitrogen retention in integrated nutrient management: assessment in a large basin by different modelling approaches. Environ. Res. Lett..

[bib23] Haines-Young R.H., Potschin M., Raffaelli D., Frid C. (2010). The links between biodiversity, ecosystem services and human well-being. Ecosystem Ecology: A New Synthesis. BES Ecological Reviews Series.

[bib24] Hecht J.E. (2007). National Environmental Accounting: A Practical Introduction. Int. Rev. Environ. Resour. Econ..

[bib25] Kadlec R.H., Knight R.L. (1996). Treatment Wetlands.

[bib26] Kadlec R.H., Wallace S.D. (2009). Treatment Wetlands.

[bib27] Kareiva, P., Tallis, H., Ricketts, T.H., Daily, G.C., Polasky, S. (Eds.), 2011. Natural Capital: Theory and Practice of Mapping Ecosystem Services, Oxford University Press, Oxford, UK.

[bib28] La Notte A., Turvani M., Giaccaria S. (2011). Economic valuation of ecosystem services at local level for policy makers and planners. The case of the island of St. Erasmo in the Lagoon of Venice. Environ. Econ..

[bib29] La Notte A., Maes J., Grizzetti B., Bouraoui F., Zulian G. (2012). Spatially explicit monetary valuation of water purification services in the Mediterranean bio-geographical region. Int. J. Biodivers. Sci. Ecosyst. Serv. Manag..

[bib30] La Notte A., Liquete C., Grizzetti B., Maes J., Egoh B., Paracchini M.L. (2015). An ecological-economic approach to the valuation of ecosystem services to support biodiversity policy. A case study for nitrogen retention by Mediterranean rivers and lakes. Ecol. Indic..

[bib31] La Notte, A., Maes, J., Thieu, V., Bouraoui, F., Masi, F., 2012b. Biophysical Assessment and Monetary Valuation of Ecosystem Services: Scenario analysis for the case of water purification in Europe, Joint Research Centre Scientific and Technical Research Series EUR 25638 EN, Publications Office of the European Union: Luxembourg, doi: 10.2788/72082.

[bib32] Maes J., Egoh B., Willemen L., Liquete C., Vihervaara P., Schagner J.P., Grizzetti B., Drakou E.G., Notte A.L., Zulian G., Bouraoui F., Luisa Paracchini M., Braat L., Bidoglio G. (2012). Mapping ecosystem services for policy support and decision making in the European Union. Ecosyst. Serv..

[bib33] Millennium Ecosystem Assessment (2005). Ecosystems and Human Well-being: Bio-diversity Synthesis (Millennium Ecosystem Assessment series).

[bib34] Mulholland P.J., Helton A.M., Poole G.C., Hall R.O., Hamilton S.K., Peterson B.J., Tank J.L., Ashkenas L.R., Cooper L.W., Dahm C.N., Dodds W.K., Findlay S.E.G., Gregory S.V., Grimm N.B., Johnson S.L., McDowell W.H., Meyer J.L., Valett H.M., Webster J.R., Arango C.P., Beaulieu J.J., Bernot M.J., Burgin A.J., Crenshaw C.L., Johnson L.T., Niederlehner B.R., O’Brien J.M., Potter J.D., Sheib-ley R.W., Sobota D.J., Thomas S.M. (2008). Stream denitrification across biomesand its response to anthropogenic nitrate loading. Nature.

[bib35] Remme R.P., Schröter M., Hein L. (2014). Developing spatial biophysical accounting for multiple ecosystem services. Ecosyst. Serv..

[bib36] Rockström J., Steffen W., Noone K., Persson Å., Chapin F.S., Lambin E.F., Lenton T.M., Scheffer M., Folke C., Schellnhuber H.J., Nykvist B., De Wit C.A., Hughes T., Van Der Leeuw S., Rodhe H., Sörlin S., Snyder P.K., Costanza R., Svedin U., Falkenmark M., Karlberg L., Corell R.W., Fabry V.J., Hansen J., Walker B., Liverman D., Richardson K., Crutzen P., Foley J.A. (2009). A safe operating space for humanity. Nature.

[bib37] Schröter M., Barton D.N., Remme R.P., Hein L. (2014). Accounting for capacity and flow of ecosystem services: a conceptual model and a case study for Telemark, Norway. Ecol. Indic..

[bib38] Sutton M.A., Howard C.M., Erisman J.W., Billen G., Bleeker A., Grennfelt P., van Grinsven H., Grizzetti B. (2011). The European Nitrogen Assessment Sources, Effects and Policy Perspectives.

[bib39] The Economics of Ecosystems and Biodiversity, 2010. The Economics of Ecosystems and Biodiversity: Mainstreaming the Economics of Nature: A Synthesis of the Approach, Conclusions and Recommendations of TEEB.

[bib40] United Nations (1993). Integrated Environmental and Economic Accounting.

[bib41] United Nations Environmental Program, 2015. United Nations Statistical Division, Convention of Biological Diversity, Norwegian Ministry of Foreign Affairs. SEEA Experimental Ecosystem Accounting: Technical Recommendations. Consultation Draft – December. Downloadable at 〈http://unstats.un.org/unsd/envaccounting/ceea/meetings/eleventh_meeting/BK-11-3b-2.pdf〉

[bib42] United Nations Statistical Division, 2003. European Commission, International Monetary Fund, Organization for Economic Co-operation and Development, World Bank. Integrated Environmental and Economic Accounting, Studies in Method, Handbooks of National Accounting (ST/ESA/STAT/SERF/Rev.1)

[bib43] United Nations, European Commission, 2014a. Food and Agriculture Organization, International Monetary Fund, Organisation for Economic Co-operation and Development, and the World Bank. System of Environmental-Economic Accounting 2012. Central Framework, Downloadable at 〈http://unstats.un.org/unsd/envaccounting/seeaRev/SEEA_CF_Final_en.pdf〉

[bib44] United Nations, European Commission, 2014b. Food and Agriculture Organization, Organisation for Economic Co-operation and Development, and the World Bank. System of Environmental-Economic Accounting 2012: Experimental Ecosystem Accounting - final, official publication, Downloadable at 〈http://unstats.un.org/unsd/envaccounting/seeaRev/eea_final_en.pdf〉

[bib45] Villamagna A.M., Angermeier P.L., Bennett E.M. (2013). Capacity, pressure, demand, and flow: a conceptual framework for analyzing ecosystem service provision and delivery. Ecol. Complex..

[bib46] Weber J.L., 2009. Land Cover Classification for Land Cover Accounting. Position Paper for the London Group Meeting. Canberra, 27–30 April 2009, Downloadable at 〈http://unstats.un.org/unsd/envaccounting/londongroup/meeting14/LG14_9a.pdf〉

