# Supplementary material for: Physical and monetary ecosystem service accounts for Europe: A case study for in-stream nitrogen retention
Source: Ecosyst Serv. 2017 Feb;23:18–29. doi: 10.1016/j.ecoser.2016.11.002 (PMC5351814; doi:10.1016/j.ecoser.2016.11.002)
Supplement: Supplementary file 1 — Supplementary material [file mmc1.docx]

## A.1: Sensitivity Analysis

Sensitivity analysis is used to demonstrate if and to which extent changes to an individual input parameter produce impacts on the model response. In fact, by changing input variables, and measuring how the outcomes are affected by that change, it is possible to analyse how sensitive the model is to the individual input variables.

In the calculation of monetary values many estimates and approximations have been undertaken: from the building and O&M costs to the discount rates and the number of years estimated as life expectancy. However, given the conceptual basis of the whole procedure, the major impact on the final result should be produced by the changes involved in the biophysical assessment rather than those depending on pure economic valuation figures. In fact, monetary valuation should just translate the outcomes of biophysical assessment. We thus need to find out the parameters that mostly affect the final economic value obtained for water purification. The purpose of the sensitivity analysis here performed is to find out how both biophysical model and economic valuation inputs affect the final output.

Firstly, we identify the variables and parameters, specifically:

1. Diffuse input sources to the river
2. Point input sources to the river
3. River retention potential (%)
4. K_20_ FWS
5. Temperature parameter Θ
6. K_20_ HF
7. Nitrogen load per PE
8. Specific hydraulic load per PE
9. Building cost FWS
10. Building cost coeff FWS
11. Building cost HF
12. Building cost coeff HF
13. O&M cost FWS
14. O&M cost HF
15. Discount rate i
16. Life expectancy N

Secondly, we randomly draw 1000 new sets of parameters from a 10% interval around the mean parameter value to study how the outputs are affected.

Thirdly, we perform a general regression as approximation of model simulation output. Sensitivity coefficients based on the proportion of total variation explained by each factor/parameter were calculated from regression fits according to the equation A.1.

$SC_{i}=\frac{SS_{i}}{TSS}*100$ Equation A.1

*where:*

*SCi: sensitivity coefficient as relative sum of squares attributable to factor I (%)*

*SSi: sum of squares for a regression model with factor i*

*TSS: total sum of squares of the output variable*

**Table A.1.** Sensitivity coefficients for the relevant variables and parameters

|  | **SSi** | **SCi** |
| --- | --- | --- |
| Diffuse input sources to the river | 85.45 | 1.02 |
| Point input sources to the river | 1399.56 | **16.671** |
| **River retention potential (%)** | 3213.42 | **38.277** |
| K20 FWS | 41.46 | 0.493 |
| **Θ** | 2179.97 | **25.966** |
| K20 HF | 7.66 | 0.091 |
| Nitrogen load per PE | 66.35 | 0.790 |
| Specific hydraulic load per PE | 1.82 | 0.021 |
| Building cost FWS | 79.57 | 0.947 |
| Building cost coeff FWS | 550.97 | 6.562 |
| Building cost HF | 15.56 | 0.185 |
| Buidling cost coeff HF | 124.47 | 1.482 |
| O&M cost FWS | 421.09 | 5.015 |
| O&M cost HF | 133.47 | 1.589 |
| Discount rate i | 24.45 | 0.291 |
| Life expectancy N | 49.88 | 0.594 |
| TSS | 8395 |  |

As reported in Table A.1, 81% of the model results are explained by variables that depend of the biophysical assessment part. Specifically, 56% depends on the model input (diffuse and point emissions to the river) and parameters (river retention). 27% depends on the physical base of the replacement cost, namely the parameters used to size the area of CW necessary to retain the amount of N (especially Θ requires particular attention). Only 17% depends on the purely economic figures, i.e. building and O&M costs and their coefficients, the discount rate and life expectancy of the CWs.

We demonstrate that the drivers of changes in the final outcome mainly depend on the biophysical assessment and results are thus consistent with the conceptual basis.
